# Relationship between the number of glaucoma medications, ocular surface disorder, and treatment adherence

**DOI:** 10.5935/0004-2749.2021-0525

**Published:** 2023-03-20

**Authors:** Gustavo A. Samico, Ricardo Y Abe, Tiago S. Prata, Sergio Henrique Teixeira, Augusto Paranhos Jr, Carolina P. B. Gracitelli

**Affiliations:** 1 Department of Ophthalmology and Visual Science, Escola Paulista de Medicina, Universidade Federal de São Paulo, São Paulo, SP, Brazil; 2 Hospital Oftalmológico de Brasília, Brasília, DF, Brazil; 3 Centro de Estudos Alcides Hirai, Ver Mais Oftalmologia, Vinhedo, SP, Brazil

**Keywords:** Medication adherence, Ocular surface disease, Ophthalmic solutions, Glaucoma, Adesão à medicação, Doenças da superfície ocular, Soluções oftálmicas, Glaucoma

## Abstract

**Purpose:**

To determine the relationship of ocular surface disease, the number of glaucoma medications prescribed and its influence on treatment adherence.

**Methods:**

In this cross-sectional study, demographic data of patients with glaucoma were collected, and patients completed the ocular surface disease index questionnaire and the glaucoma treatment compliance assessment tool. Ocular surface parameters were assessed by “Keratograph 5M.” Patients were stratified into two groups according to the amount of prescribed ocular hypotensive eye drops (Group 1, one or two classes of medications; Group 2, three or four classes)

**Results:**

In total, 27 eyes of 27 patients with glaucoma were included: 17 using 1 or 2 topical medications (Group 1) and 10 eyes using 3 or 4 classes (Group 2). For the Keratograph assessment, patients using ≥3 medications had significantly smaller tear meniscus height (0.27 ± 0.10 vs. 0.43 ± 0.22; p=0.037). The analysis of Ocular Surface Disease Index questionnaire showed higher scores among the groups using more hypotensive eye drops (18.67 ± 13.53 vs. 38.82 ± 19.72; p=0.004). Regarding the glaucoma treatment compliance assessment tool, Group 2 had worse scores in components of forgetfulness (p=0.027) and barriers due to lack of drops (p=0.031).

**Conclusion:**

Patients with glaucoma using more hypotensive eye drops had worse tear meniscus height and ocular surface disease index scores than those using fewer topical medications. Patients using three or four drug classes had worse predictors of glaucoma adherence. Despite worse ocular surface disease results, no significant difference in self-reported side effects was found.

## Abstract (pt)

**Objetivo:**

Determinar a relação entre doença da superfície ocular (OSD), número de medicamentos prescritos para o glaucoma, e como isso influencia na adesão ao tratamento.

**Métodos:**

Neste estudo transversal, pacientes com glaucoma foram submetidos à coleta de dados demográficos, preenchimento do questionário *Ocular Surface Disease Index* e do *Glaucoma Treatment Compliance Assessment Tool*. Os parâmetros da superfície ocular foram avaliados pelo “Keratograph 5M”. Indivíduos foram estratificados em 2 grupos de acordo com a quantidade de colírios hipotensores oculares prescritos (Grupo 1: uma ou duas classes de medicamentos; Grupo 2: três ou quatro classes).

**Resultados:**

No total, 27 olhos de 27 pacientes com glaucoma foram incluídos: 17 usando 1 ou 2 medicamentos tópicos (Grupo 1) e 10 olhos usando 3 ou 4 classes (Grupo 2). Na avaliação do Keratograph, os pacientes em uso de 3 ou mais medicamentos apresentaram altura do menisco lacrimal significativamente menor (0,27 ± 0,10 vs. 0,43 ± 0,22; p=0,037). Análise do questionário OSDI mostrou escores mais altos entre o grupo que usou mais colírios hipotensores (18,67 ± 13,53 vs. 38,82 ± 19,72; p=0,004). Em relação ao *Glaucoma Treatment Compliance Assessment Tool,* o Grupo 2 apresentou piores escores nos componentes de esquecimento (p=0,027) e barreiras por falta de colírios (p=0,031).

**Conclusão:**

O estudo demonstrou que pacientes com glaucoma usando mais colírios hipotensivos apresentaram piores escores de altura do menisco lacrimal e *Ocular Surface Disease Index*, em comparação com aqueles que usaram menos medicamentos tópicos. Pacientes em uso de 3 ou 4 classes de colírios tiveram piores preditores de adesão ao glaucoma. Apesar dos piores resultados de doença da superfície ocular, não houve diferença significativa nos efeitos colaterais relatados.

## INTRODUCTION

Glaucoma is one of the leading causes of irreversible vision loss worldwide^(1)^. The most common treatment includes self-administered topical hypotensive eye drops to slow down progressive retinal ganglion cell damage and prevent vision loss^(2)^. Thus, medical adherence is fundamental to maximizing the benefits of therapy^(3)^. However, patients tend to deviate from the prescribed medical regimen, with an average nonadherence rate of 40%^(4)^, leading to greater visual field loss^(5)^.

Compliance with glaucoma treatment is a parameter that is complex and difficult to measure. Several studies have assessed the barriers and found that nonadherence was associated with forgetfulness, difficulty with drop application, lack of knowledge about the disease, and being out of drops^(6,7,8)^. Based on the constructs of the health belief model, Mansberger et al. developed a questionnaire called glaucoma treatment compliance assessment tool (GTCAT)^(9)^. This model postulates that patients value health, consider disease as a threat with avoidable consequences, and expect positive outcomes of treatment^(10)^. One of reasons for poor glaucoma adherence is the side effects of treatment ^(11)^.

Ocular surface disease (OSD) is a pathology frequently related to glaucoma because dry eye symptoms were reported by 59% of patients, of which 27% describe severe symptoms^(12)^. Compared with the control group, the glaucoma group also showed higher OSD index (OSDI) scores and worse objective parameters of OSD^(13)^.

The etiology of OSD has been associated with the chronic use of intraocular pressure (IOP)-lowering therapies^(14)^. Both the active principle of ocular hypotensive eye drops and the preservative used, usually benzalkonium chloride (BAK), were found to cause and/or aggravate changes in the ocular surface^(15)^.

This study aimed to investigate hallmarks of OSD, measured using subjective (e.g., OSDI questionnaire) and objective (e.g., Keratograph and clinical analysis) parameters, its relationship between the number of glaucoma drugs prescribed, and how it influences treatment adherence assessed through GTCAT.

## METHODS

### Participants

This observational cross-sectional study included volunteers who had a confirmed diagnosis of glaucoma using at least one hypotensive eye drop for the last 6 months and were selected from the glaucoma outpatient clinic of *Hospital São Paulo* at *Universidade Federal de São Paulo*. Written informed consent was obtained from all participants. This study was approved by the Institutional Review Board of the Federal University of São Paulo (No. 1022/2019) and the methodology adhered to the tenets of the Declaration of Helsinki.

Glaucoma was diagnosed based on the presence of repeatable (≥2 consecutive) abnormal standard automated perimetry test results using the 24-2 Swedish Interactive Threshold Algorithm Standard program of the visual field (Humphrey Field Analyzer; Carl Zeiss Meditec, Inc.). An abnormal visual field was determined by the presence of a pattern standard deviation with p<0.05 and/or glaucoma hemifield test result outside normal limits. The participants were considered to have glaucoma if at least one eye had a repeatable and reliable glaucomatous visual field defect.

For standardization, the right eye was always used as a reference, except when the exclusion criteria were met, which led to the analysis of the left eye. The exclusion criteria were as follows: (i) systemic diseases or oral medications that affect the ocular surface; (ii) acute diseases that affect the ocular surface; (iii) previous ocular trauma or surgery, except for phacoemulsification; and (iv) use of contact lenses. The enrolled participants were stratified into two groups according to the number of topical IOP-lowering medications (Group 1: one or two classes of medications; Group 2, three or four classes of medications). All patients were using free samples of drugs provided by the healthcare system. No patients were using a fixed combination or preservative-free eye drops.

### Clinical evaluation

All patients underwent a comprehensive ophthalmologic examination, including a review of the medical history, best-corrected visual acuity, slit-lamp biomicroscopy, and Goldmann applanation tonometry. Signs of OSD were assessed using the tear break-up time (BUT), which was classified as altered if <5 s. Bulbar redness (BR) was scored 0-4 according to the Institute for Eye Research-Brien Holden Vision Institute scales^(16)^ using comparative photographs of BR. Signs of keratitis were also evaluated by staining the corneal surface with fluorescein eye drops and then classified as absent or present (slight, moderate, or severe).

### Demographic and socioeconomic parameters

Socioeconomic and clinical questionnaires were also administered to the patients. These questionnaires contained a survey about demographics, history of ocular and systemic conditions, educational level (at least high school degree, yes/no), and systemic comorbidities. The number of topical antiglaucoma medications was identified, and the use of prostaglandins was classified as yes or no.

### Keratograph analysis

Ocular surface objective parameters were assessed by the Keratograph 5M (Oculus, Wetzlar, Germany). Noninvasive tear BUT (NITBUT) was measured three times for the reference eye using and infrared (IR) video tool. The NITBUT-first (time in seconds of the first tear break-up) was generated for each measure, and a simple average was calculated to obtain the results. The BR was graded automatically by an anterior biomicroscopy photograph of the Keratograph. The tear meniscus height (TMH) was evaluated once using IR images from the Oculus TMH tool. It was perpendicular to the lid margin central point, relative to the pupil center (in millimeters). Meibography was also performed by upper eyelid eversion using IR-light and IR-sensitive camera to visualize the meibomian glands. The images were graded manually from 0 to 3 using the Jenvis grading scale (grade 0, no gland loss; grade 1, area of loss smaller than 1/3; grade 2, loss between 1/3 and 2/3; and grade 3, area of loss greater than 2/3)^(17)^.

### OSDI

The presence and severity of OSD symptoms were evaluated using the OSDI questionnaire. This tool was validated in Brazil by Prigol et al.^(18)^, and it includes 12 questions that are divided into three subscales: (1) related to visual function (questions 1-5), (2) associated with ocular symptoms (questions 6-9), and (3) regarding environmental triggers (questions 10-12). Individual OSDI questions were scored from 0 to 4, with scores of 0, 1, 2, 3, and 4 corresponding to answers of none, some, half, most, and all of the time, respectively. A total score was calculated using the following equation: 25 × [(sum of individual question scores) / (number of questions answered)], yielding a total score ranging from 0 to 100^(19)^.

### GTCAT

To assess adherence to glaucoma treatment, the GTCAT^(9)^ (short version, v2019.1, Copyright 2019, Legacy Health System) was administered. The GTCAT is validated in Brazil by Abe et al.^(20)^, and it includes 27 statements developed from constructs of the health belief model, expert opinion, and previous studies regarding treatment compliance in patients with glaucoma^(10)^. Responses of questionnaire statements are reported in a 5-interval Likert-type scale using “disagree” or “agree” (e.g., 1, disagree a lot; 5, agree a lot). The Brazilian Portuguese version could find seven different components of treatment adherence: self-efficacy, experience of the negative effects of glaucoma, well-being, general glaucoma knowledge, glaucoma symptom knowledge, cues to action, and barriers due to lack of drops.

### Statistical analysis

The descriptive analysis included the mean and standard deviation for variables with a normal distribution, whereas variables that were not distributed normally were presented as the median and interquartile range. The skewness-kurtosis test and histograms were used to check for normality. The t-test was used for multiple comparisons between groups, and for non-normal variables, the corresponding nonparametric test (Wilcoxon rank test) was performed. All statistical analyses were performed using Stata (StataCorp LP, College Station, TX). The α level (type I error) was set at 0.05.

## RESULTS

This study evaluated 27 eyes of 27 patients with glaucoma between June 2019 and January 2020: 17 patients were using one or two topical hypotensive medications (Group 1), whereas 10 were using three or four hypotensive eye drops (Group 2). The mean age was comparable in both groups (73.47 ± 9.vs. 66.6 ± 18.52 years, respectively; p=0.279). No significant difference in sex was found between the groups (p=0.260). The visual acuity in Groups 1 and 2 (0.39 ± 0.29 vs. 0.86 ± 0.87 logMAR, p=0.335) was comparable. Group 2 presented with lower visual field mean deviation (MD) than Group 1 (- 14.61 ± 3.12 vs. - 6.12 ± 1.38, respectively; p=0.035). Table 1 summarizes the demographic and clinical findings of our study.

**Table 1:** Demographic and clinical findings

	Group 1 (n=17)	Group 2 (n=10)	p-value
Age (years)	73.47 ± 9.51	66.66 ± 18.52	0.279
Sex (%)			0.260
Female	10 (58.82)	8 (80.0)	
Male	7 (41.18)	2 (20.0)	
Race (%)			0.201
Black	3 (17.65)	4 (40.0)	
Other	14 (82.35)	6 (60.0)	
IOP (mmHg)	13.88 ± 3.50	14.4 ± 3.95	0.665
Visual acuity of the eye of reference (logMAR)	0.39 ± 0.29	0.86 ± 0.87	0.335
VF MD eye of reference (dB)	-6.12 ± 5.55	-14.60 ± 9.88	0.035
Prostaglandin use (yes, %)	13 (76.47)	10 (100)	0.097
Level of education, (> High school,%)	6 (35.29)	3 (30.0)	0.756
Marital status (married, yes %)	9 (52.94)	3 (30.0)	0.686
OSDI (units)	18.67 ± 13.53	38.82 ± 19.72	0.017

Mean (± SD).

**Figure 1:**
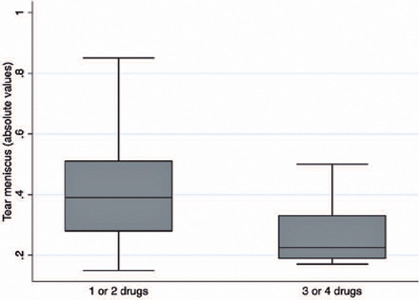
Boxplots of the distribution of the tear meniscus height among the groups. Box: median and interquartile range. Boxplot with whiskers with maximum and minimum 1.5 IQR.

**Figure 2:**
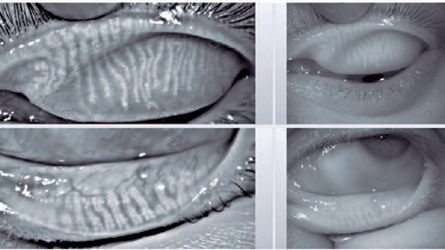
Meibography of a patient in group 1, with atrophy of less than 1/3 of the meibomian glands.

The clinical parameters of OSD were not significantly different between the groups, including keratitis (present in 5.88% vs. 20%, p=0.260), BUT (altered in 47.06% vs. 40%, p=0.722), and conjunctival hyperemia (1 point in 70.59% vs. 60%, 2 points in 0% vs. 10%, p=0.405). For the Keratograph assessment, patients with glaucoma with ≥3 medication classes had significantly smaller TMH than those using 1 or 2 drugs (0.27 ± 0.10 vs. 0.43 ± 0.22; p=0.037). Figure 1 shows the distribution of TMH between the two groups. No significant difference in NKBUT (p=0.243) and BR (p=0.314) was found. Table 2 summarizes the different parameters of OSD. Group 1 had worse meibography grades than Group 2 (1.88 ± 0.86 vs 1.00 ± 0.66; p=0.015). Figure 2 illustrates a case from Group 1, in which meibography shows atrophy of less than 1/3 of the meibomian glands.

**Table 2:** Keratograph analysis of the entire sample

	Group 1 (n=17)	Group 2 (n=10)	p-value
Tear meniscus height (mm)	0.43 ± 0.22	0.27 ± 0.10	0.037
Bulbar redness (crosses)	1.96 ± 0.44	2.20 ± 0.79	0.435
Meibography (degree)	1.88 ± 0.86	1.00 ± 0.66	0.015
NITBUT (seconds)	9.29 ± 4.24	11.73 ± 6.37	0.366

NITBUT= non-invasive keratograph tear BUT.

Mean (± SD).

**Figure 3:**
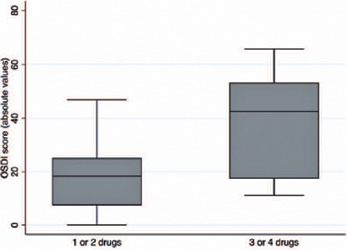
Boxplots of the distribution of the ocular surface disease index in both groups. Box: median and interquartile range. Boxplot with whiskers with maximum and minimum 1.5 IQR.

**Figure 4:**
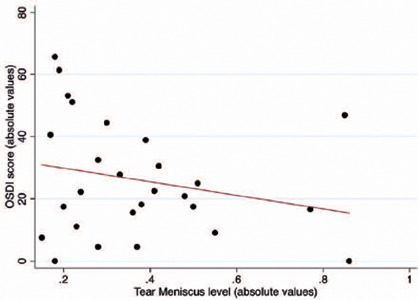
Association between the OSDI scores and the tear meniscus height. Box: median and interquartile range. Boxplot with whiskers with maximum and minimum 1.5 IQR.

In the analysis of the OSDI questionnaire, participants using three or four hypotensive eye drops had higher scores than those using one or two (18.67 ± 13.53 vs. 38.82 ± 19.72; p=0.017). Figure 3 shows the distribution of the OSDI scores. Higher OSDI scores were also associated with smaller TMH (r=0.237; p=0.018). Figure 4 represents the association between OSDI and TMH.

**Figure 5:**
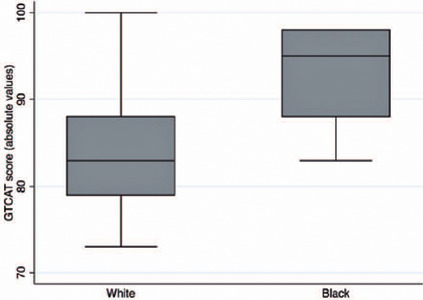
Boxplots of the distribution of Glaucoma Treatment Compliance Assessment Tool (GTCAT) scores in white and black patients. Box: median and interquartile range. Boxplot with whiskers with maximum and minimum 1.5 IQR.

Regarding the GTCAT, Group 2 had worse scores in components of forgetfulness (p=0.027) and barriers due to lack of drops (p=0.031) than Group 1. Both groups showed comparable results on other constructs of the questionnaire. Higher GTCAT scores were also significantly associated with race (p=0.004), suggesting that black patients may have better predictors of treatment adherence. Figure 5 shows the distribution of GTCAT in white and black patients.

## DISCUSSION

This study found that patients with glaucoma using three or more hypotensive eye drops had worse objective and subjective OSD parameters than those using one or two topical medications, including worse TMH evaluated by the Keratograph and worse OSDI scores. Patients using more classes of drugs also showed worse predictors of glaucoma adherence in the constructs of the GTCAT questionnaire related to forgetfulness and barriers to treatment.

In our population, patients with glaucoma using more or fewer drugs did not present with a significant difference in the clinical parameters of the ocular surface, including BR, fluorescein BUT, and presence of keratitis. These results may be explained by the small sample of individuals and the nature of these variables, which can only be classified in crosses or present/absent. Moreover, we did not use Lissamine green or Rose Bengal, which are vital dyes that could provide a better assessment of devitalized cells. Previous studies have found that patients with glaucoma using topical medications had worse keratitis, BR^(13)^, and BUT^(21)^ than a control nonglaucomatous population. Since both of our study groups were chronically using hypotensive eye drops (and its preservative), probable differences between them might be subtle in clinical examination, requiring a larger sample to obtain the expected statistical results.

Objective data acquired from Keratograph 5M have shown a significantly lower TMH in the group using a higher number of glaucoma medications. In the reviewed literature, lower TMH also correlated with greater cumulative preservative concentration^(22)^. NITBUT and BR were not useful in discriminating the amount of the prescribed hypotensive eye drops in our population. Other authors have found similar results, i.e., the NITBUT was not associated with the number of glaucoma eyedrops used per day^(22,23)^, although BR had shown a positive association with the number of drops^(23)^. This may be due to the lack of specificity of the NITBUT test, which can be altered by other individual factors^(18)^. Surprisingly, patients using one or two medications had worse meibography grades than those using three or four classes of drugs. Previous studies have not reported an association between the number of hypotensive eye drops and atrophy of the meibomian glands^(23,24)^, despite patients with glaucoma having worse scores than the control group^(13,24)^. Prostaglandin analogs are associated with the obstructive type of meibomian gland dysfunction^(25)^. We could not find this relationship, possibly because almost all of our patients were using this type of medication (including the group using fewer medications), compromising the comparison with individuals who did not use this drug class. The duration of topical therapy was also not considered. Prospective studies of participants beginning treatment with antiglaucoma eye drops should clarify this contradictory finding.

Study results have shown significantly higher OSDI scores in the group using more hypotensive medications, which means that a higher number of drugs was associated with more symptoms. Some authors could confirm the correlation between the number of prescribed glaucoma medications and ocular surface symptoms^(25,26)^, whereas others were unable to reach this finding^(12,22)^. Glaucoma treatment increases the risk of OSD compared with normal subjects^(12,13,14,15,22,23,24)^. This link has an important clinical role because dry eye disease has been extensively associated with the quality of life of patients with glaucoma^(26)^. A significant correlation was also found between the OSDI score and TMH in our studied population, confirming the hypothesis that a smaller TMH results in worse OSD symptoms. In the present study, other objective parameters, such as BR and NITBUT, did not correlate with the OSDI questionnaire scores. In two previous studies, corneal staining most strongly correlated with OSDI scores in patients with glaucoma, and NITBUT also showed no influence in the questionnaire results. They suggested that tear instability can no longer produce further symptoms beyond a certain point of the treatment burden. The controversial correlation between signs and symptoms of OSD may be explained by BAK mechanism of decreasing corneal nerve density, leading to reduced corneal sensitivity. Thus, glaucoma experts should actively search for both signs and question OSD symptoms at every opportunity. Treating dry eye disease in patients with glaucoma can improve the OSDI score, best-corrected visual acuity, and acute BR and may have a role in reducing the IOP^(27)^.

The group using a higher number of eye drops had lower GTCAT scores in two specific constructs related to adherence, namely, forgetfulness and barriers due to lack of drops. Both groups had similar results related to knowledge, self-efficacy, susceptibility, cues to action, and side effects. Forgetfulness is a major cause of non-intentional nonadherence in patients with glaucoma^(6,7,28,29)^. This suggests that our population may benefit from reminders and schedules to improve medical compliance. Barriers due to lack of drops were also prevalent in some studies^(7,28,29)^, pointing out the importance of better healthcare systems and patient education to prevent vision loss. Although both constructs were related to the group using more hypotensive eye drops, the cross-sectional study design cannot imply any causality in this association. Clinicians may be adding more drugs to patient’s regimen because of the lack of adherence or individuals can be expressing more non-compliance barriers as the number of prescribed drugs increases. Other authors could not find direct correlation between the number of medications prescribed and overall treatment adherence^(3,6)^. Although patients using three or four hypotensive eye drops had worse OSDI scores and lower TMH, the GTCAT construct of side effects were not statistically different in this population. Side effects have been identified as a cause of non-compliance by interviewers^(11)^; however, recent studies could not confirm this association^(6,28)^. We may hypothesize that in some patients, the perceived dry eye symptoms do not correlate to the drugs used or maybe our population assumed that the benefits of the therapy overcome its unpleasant side effects. In the reviewed literature, other frequently cited obstacles to adherence include depression^(5,29)^, knowledge about glaucoma^(8,29)^, and self-efficacy^(6,28,29)^. Interestingly, this study showed that black patients had higher GTCAT scores, which indicated that they were more adherent to treatment. This contradicts previous data that related the black population to worse glaucoma treatment compliance^(3,5,8)^. In this study, only a small sample of a mixed-race country was assessed, leading to a higher complexity analysis and possible confounders. Moreover, the GTCAT was not designed for the use of its overall score, requiring further analysis to confirm this finding. Multicentric research from different populations is necessary to better understand this association. Intervention should aim at these multiple barriers to exert a positive effect on lasting treatment adherence, and specific factors must be identified in the early stage of the disease. A coaching program combining motivational interviews, reminder systems, and tailored education was found to improve glaucoma medication adherence^(30)^.

This study has limitations. First, this is a single-center cross-sectional study with a small sample size. Second, we did not measure the relative humidity of the examination room. Third, we did not consider the cumulative dose of medications, time of drug exposure, and use of preservatives and eye lubricants. Fourth, the use of reference, similar or generic drugs, was also not considered. Fifth, patients were not stratified according to classes of medications, besides prostaglandin use. However, this study provided valuable information on adherence, dry eye disorder in the study population, using the OSDI questionnaire, and objective parameters from Keratograph and GTCAT.

In conclusion, this study found that patients with glaucoma using more hypotensive eye drops had worse TMH and OSDI scores than those using fewer topical medications. Patients using three or four classes of drugs also showed worse predictors of glaucoma adherence related to forgetfulness and barriers due to lack of eye drops. Despite worse OSD results, no significant difference in self-reported side effects as a compliance barrier was found. A larger sample is needed to determine the weight of these factors in treatment adherence.
